# Generation of a predicted protein database from EST data and application to iTRAQ analyses in grape (*Vitis vinifera *cv. Cabernet Sauvignon) berries at ripening initiation

**DOI:** 10.1186/1471-2164-10-50

**Published:** 2009-01-26

**Authors:** Joost Lücker, Mario Laszczak, Derek Smith, Steven T Lund

**Affiliations:** 1 Wine Research Centre, Faculty of Land and Food Systems, University of British Columbia, Vancouver, BC, Canada; 2 Bioinformatics Group, Canada's Michael Smith Genome Sciences Centre, BC Cancer Agency, Vancouver, BC, Canada; 3 University of Victoria-Genome British Columbia Proteomics Centre, Victoria, BC, Canada

## Abstract

**Background:**

iTRAQ is a proteomics technique that uses isobaric tags for relative and absolute quantitation of tryptic peptides. In proteomics experiments, the detection and high confidence annotation of proteins and the significance of corresponding expression differences can depend on the quality and the species specificity of the tryptic peptide map database used for analysis of the data. For species for which finished genome sequence data are not available, identification of proteins relies on similarity to proteins from other species using comprehensive peptide map databases such as the MSDB.

**Results:**

We were interested in characterizing ripening initiation ('veraison') in grape berries at the protein level in order to better define the molecular control of this important process for grape growers and wine makers. We developed a bioinformatic pipeline for processing EST data in order to produce a predicted tryptic peptide database specifically targeted to the wine grape cultivar, *Vitis vinifera *cv. Cabernet Sauvignon, and lacking truncated N- and C-terminal fragments. By searching iTRAQ MS/MS data generated from berry exocarp and mesocarp samples at ripening initiation, we determined that implementation of the custom database afforded a large improvement in high confidence peptide annotation in comparison to the MSDB. We used iTRAQ MS/MS in conjunction with custom peptide db searches to quantitatively characterize several important pathway components for berry ripening previously described at the transcriptional level and confirmed expression patterns for these at the protein level.

**Conclusion:**

We determined that a predicted peptide database for MS/MS applications can be derived from EST data using advanced clustering and trimming approaches and successfully implemented for quantitative proteome profiling. Quantitative shotgun proteome profiling holds great promise for characterizing biological processes such as fruit ripening initiation and may be further improved by employing preparative techniques and/or analytical equipment that increase peptide detection sensitivity via a shotgun approach.

## Background

The field of protein discovery through mass spectrometry (MS) continues to grow rapidly but the number of species for which finished (i.e. > 98% complete) whole genome sequence data are available is currently not keeping pace. For a large number of laboratories worldwide studying proteomes in 'non-mainstream' organisms, annotations of tandem mass spectra data must rely on open reading frame (ORF) predictions from expressed sequence tag (EST) data from their species of interest or a phylogenetically close relative. ESTs generated from single pass sequencing reactions are frequently not full length and the reading frames are unknown. Proteolytic peptide sequence databases derived from multiple, truncated predicted ORFs per each of thousands of ESTs can hamper the ability of search engines such as MASCOT [1] and algorithms such as Paragon in ProteinPilot software [2] to make statistically robust protein identifications from MS/MS spectrum data [3]. Protein identifications from MS/MS spectra may be further complicated when the EST data that are used to build a peptide sequence database are created based on one genotype for a given species. We report here on the development of scripts for the generation of a predicted tryptic peptide sequence database based on EST data in grapevine. Our computational approach accounts for multiple open reading frames, truncated predicted ORFs, and the presence of N-terminal signal peptides, and may be useful for MS/MS-based protein discovery in any species for which EST data are available.

Quantitative protein expression profiling analyses in plants have increasingly implemented stable isotopic labeling as an advance or complement to two dimensional gel electrophoresis (2DGE) methods. Isotope coded affinity tagging (ICAT) reagents are used to covalently label cysteine residues with heavy or light hydrogen or carbon in two complex peptide samples, for example, wild type versus mutant genotypes. The ICAT chemistry is used to purify labeled peptides via affinity chromatography and then samples are mixed and subjected to LC-MS/MS [4]. One of the first reports on an ICAT application in plants was in wheat (*Triticum aestivum *L.) where relative expression in monosomic deletion mutants was used to begin to clarify the influence of ancestral genomes on differential seed protein expression for breeding applications [5]. The ICAT technique is limited, however, by the tagging of cysteine residues only, as well as the need for affinity purification of labeled peptides; invariably, information is lost through these steps. An improvement to the ICAT technique involves the labeling of amine groups using a set of four or more isobaric tags. The advantages of this technique, isobaric tagging for relative and absolute quantitation (iTRAQ), are that most peptides are labeled, no affinity purification step is required, and the isobaric nature of the tags allows co-elution of identical peptides that are differentially tagged, thereby enriching detection sensitivity and accuracy in comparison to ICAT [6]. Few reports of iTRAQ implementation in plant proteome studies have been reported but pioneering work in this field has been successful, for example, in further defining the organellar proteome in Arabidopsis [7], characterizing pathogen defense mechanisms in Arabidopsis [8], and clarifying micronutrient stress responses in barley (*Hordeum vulgare*) [9].

We were interested in characterizing ripening initiation in grape berries at the level of differential protein expression in order to better define the molecular control of this important process for grape growers and wine makers. Grape berry ripening is non-climacteric and ethylene does not act as a major signal initiating this process, as it does in climacteric species such as tomato (*Lycopersicon esculentum*). Abscisic acid (ABA), hexoses, and brassinosteroids (BRs) have previously been implicated in non-climacteric ripening regulation but how these and potentially other signaling pathways interact to effect major changes in berry biochemistry at ripening initiation is poorly understood. The tissues in the grape berry consist of the seeds, the mesocarp (flesh), and the exocarp (skin); the pericarp refers to the mesocarp and exocarp, collectively. Primary and secondary compounds important for grape and wine products begin to accumulate in the exocarp and/or the mesocarp at ripening initiation [10], so we considered that it was important to evaluate changes in the berry proteome separately in these tissues. To date, a limited number of reports on proteome profiling in grapevine and grape berries have been published in which 2DGE was employed [11-14]. We considered that the iTRAQ technique could be useful in surmounting some technical limitations encountered with 2DGE and allow us to detect a greater number of proteins per sample. In this report, we demonstrate the application of our computational approach to tryptic peptide sequence database development from a large collection of grapevine EST data and validate its usefulness by showing improved detection and annotations of MS/MS data derived from grape exocarp and mesocarp total protein extracts. We further provide new quantitative information on differential protein expression during ripening initiation in grape berries. This is the first report in which iTRAQ has been used to study differential protein expression in any fruit.

## Methods

### Plant material

Grape clusters were sampled from *V. vinifera *cv. Cabernet Sauvignon clone 15 grafted on rootstock 101-14 in a commercial vineyard near Osoyoos, British Columbia, in the 2004 and 2005 seasons. Sampling dates during each season were focused on the developmental stages undergoing ripening initiation. Clusters were sampled on a single date in 2004, August 12^th^, which was the timing of approximately 50% ripening initiation based on a turning pink color phenotype. For the 2005 season, the ripening initiation stage was sampled over a longer period (August 10^th ^through August 16^th^), since in this growing season, ripening advanced slowly due to lower atmospheric temperatures. Five clusters from five different vines were sampled in each season and snap frozen directly in liquid nitrogen in the vineyard and then transported on dry ice to UBC Vancouver where they were stored at -80°C.

Individual grapes from each of the 2004 and 2005 clusters were developmentally staged based on a visual pigmentation assessment and were segregated for each season into green, pink/turning, fully turned red, and fully turned purple phenotypic classes. For the 2005 samples, green grapes were only taken from clusters collected on August 10^th^, since for this date and August 12^th ^there was no visible change in color present in any of the grape clusters. Thirty grapes of similar sizes per pigmentation class per year were segregated for experimentation. Prior to total protein extraction, individual grapes were partially thawed in gloved hands and then, using a forceps, the exocarp tissue was carefully peeled away from the mesocarp and placed immediately into liquid nitrogen. Seeds were then carefully removed while keeping the remaining mesocarp tissue frozen in liquid nitrogen. Exocarp and mesocarp samples were ground to a powder under liquid nitrogen and then used for total protein extractions.

### Tissue preparation for protein extraction

Preparation of exocarp tissue samples for protein extraction was performed according to a previously described protocol for olive leaf [15] with some modifications described here. The procedure was carried out on ice and centrifugations were performed at 4°C. Throughout the procedure, each wash was done by complete resuspending of the tissue pellet. Four hundred mg of powdered exocarp tissue was placed in a 2 mL G-tube (Fisher Scientific Canada, Ottawa, ON). The tissue was suspended in 1.5 mL of a cold (-20°C) ethyl acetate:ethanol (1:2 (v:v)) solution by vortexing for 30 s; the ethyl acetate:ethanol extraction was previously found to be useful for removing pectins as well as pigments such as chlorophylls [16]. Following centrifugation for 3 min at 21000 × g, the supernatant was removed and the ethyl acetate:ethanol extraction and centrifugation steps were repeated on the remaining tissue. The sample was next extracted twice with cold (-20°C) 100% acetone by vortexing and centrifuging, as before. Subsequently, the tissue with added acetone was transferred from the G-tube to a mortar using a 1 mL pipette with the tip end excised to increase diameter and then the acetone was evaporated from the tissue at room temperature. After the addition of 1/3 vol of white quartz sand (Sigma-Aldrich, Oakville, ON, Canada) to the tissue, it was ground to an even finer powder. The powder was transferred back to a clean 2 mL G-tube by suspending the tissue in 1.5 mL of cold (-20°C) TCA:acetone (1:9 (v/v)) and vigorously mixed and centrifuged, as before. Extraction with 10% TCA:acetone was repeated five to seven times, or until no more anthocyanins (red-pigmented flavonoids) could be extracted from the tissue. This was followed by three washes with chilled (4°C) 10% TCA in water by vigorous mixing and centrifugation, as before, to extract the pectins and remaining anthocyanins from the tissue. After this, the tissue was washed twice with cold (-20°C) 80% acetone and centrifuged, as before. Protein extraction was performed after drying the tissue pellet to completion in a speed vacuum extractor (SPD131DDA, Thermo Scientific, Milford, MA, USA).

For the preparation of the mesocarp tissue, the same procedure for the exocarp was used with the following modifications. Three g of starting material was used per sample and the first extractions up to the grinding step with white quartz were done in 50 mL Oakridge tubes. Since some protein can be extracted from the mesocarp via TCA:acetone extraction alone [14], a 20 min incubation time at -20°C was introduced after the first 100% acetone step and included in the subsequent TCA:acetone containing steps to ensure that all of the protein remained precipitated. In the TCA:H_2_O step, the 20 min incubation was done on ice. Since no anthocyanins are present in mesocarp, only two TCA:acetone extractions were carried out for the mesocarp tissue.

### Total protein extraction

Two hundred to 300 mg of pre-extracted and dried exocarp or mesocarp tissue contained in a 2 mL G-tube was extracted by resuspending the pellet in 0.75 mL cold Tris-buffered phenol, pH 7.9. Then, 0.75 mL of dense SDS buffer (30% sucrose, 2% SDS, 0.1 M Tris-HCL, pH 8.0) was added. The mixture was vortexed for 30 s and incubated on ice for 40 min with intermittent vortexing. The phenol phase containing the protein as the top phase was separated by centrifugation at 21000 × g for 5 min and transferred into a clean 2 mL G-tube. The remaining SDS phase was re-extracted with another 0.75 mL Tris-buffered phenol and incubated for 20 min before centrifuging and subsequent transfer and combination of the two phenol phases. Protein was precipitated by adding a minimum of 5 vol cold methanol plus 0.1 M ammonium acetate to the combined phenol phase. Precipitation was carried out at -20°C for 30 min or overnight. After centrifugation at 21000 g for 10 min, the pellet was washed twice with cold methanol containing 0.1 M ammonium acetate and subsequently with 80% acetone twice. Pellets were next dissolved in 200–300 μL fresh buffer containing 6 M urea, 2% CHAPS, 5 mM EDTA, and 30 mM HEPES, pH 8.1, to obtain a concentration of approximately 1.0 μg/μL. Careful sonication on ice was used to dissolve the samples.

Protein quantitation was done using a bicinchoninic acid (BCA) absorption assay (Sigma-Aldrich Canada Ltd., Oakville, ON) and read in a Victor V plate reader (PerkinElmer Life and Analytical Sciences, Woodbridge, ON, Canada) equipped with a photometric filter of 560 nm and 10 nm bandwidth. The quality of each protein sample was checked via SDS-PAGE; all samples were devoid of indications of degradation and showed good resolution with low background. Total protein samples were then shipped on dry ice to the University of Victoria-Genome BC Proteomics Centre in Victoria, BC, for iTRAQ analyses. Using a second BCA assay, each protein sample was re-quantified just before aliquoting 100 μg of each sample for iTRAQ labeling steps.

### Experimental design and labeling of peptides with iTRAQ reagents

The experimental design consisted of the four developmental stages described earlier for each of exocarp 2004, mesocarp 2004, exocarp 2005, and mesocarp 2005. Two biological replicates were employed for each stage and tissue for the 2005 samples, whereas one 2004 sample was used for each stage of mesocarp or exocarp. An additional technical replicate was carried out for exocarp 2004, representing separate iTRAQ labeling reactions and analyses starting from the same protein sample.

Labeling of peptides with iTRAQ reagents (Applied Biosystems Canada, Streetsville, ON) was performed according to the manufacturer's recommendations as follows. One hundred μg of each protein sample in a maximum volume of 200 μL was precipitated overnight using 100% acetone and dissolved in 20 μL of denaturing buffer containing 1 μL denaturant and 2 μL reducing reagent (TCEP) as provided in the iTRAQ kit, followed by vortexing and incubation at 60°C for 1 h. One μL of cysteine blocking solution (MMTS) was then added to each sample, followed by incubation at room temperature for 10 min. These protein samples were digested overnight with trypsin (Promega, Madison, WI, USA) at 37°C. iTRAQ labeling was carried out by adding iTRAQ reagents 114, 115, 116, and 117 to either the exocarp or the mesocarp samples representing the four developmental stages, green, pink/turning stage, red/fully turned, and purple, respectively. Subsequently, these four samples were mixed by vortexing and further incubated at room temperature for 1 h.

The four iTRAQ-labeled peptide samples were pooled together, diluted 1:10 with cation exchange sample buffer (A) containing 25% acetonitrile in 10 mM KH_2_PO_4_, and then adjusted to pH 3.0 using phosphoric acid. Because of this acidification step, it is important to remove pectins prior to total protein extractions; we found in previous trials that the pectins likely polymerized and precipitated out of solution, converting samples mostly to a gelatinous state unsuitable for further analyses (data not shown). The combined peptide mixture was fractionated by strong cation exchange (SCX) chromatography on a BioCAD workstation (Applied Biosystems), using a 4.6 mm × 20 cm polysulfoethyl aspartamide column (PolyLC Inc, Columbia, MD, USA). First, the mixed samples were loaded in buffer A at a flow rate of 0.2 mL/min. Once completely loaded, the column was washed for 20 min with buffer A. Peptides were eluted by a linear gradient of 0 to 350 mM KCl in buffer B (20 mM KH_2_PO_4_, 25% acetonitrile, pH 3.0). Sixty-nine fractions were collected over the course of 70 min at a flow rate of 1 mL/min. Of these fractions, only 12 fractions containing the eluted labeled peptides as measured by optical density monitoring at 214 nm were chosen for analysis on a 2 h LC-MS/MS program. The fractionated samples were reduced to 150 μL in a speed-vac (Thermo-Savant, Holbrook, NY, USA) and transferred to autosampler tubes (LC Packings, Amsterdam, The Netherlands).

### Liquid chromatography and mass spectrometry

The samples were analyzed for identification and quantitation on a QSTAR Pulsar i hybrid tandem mass spectrometry (LC-MS/MS) system (Applied Biosystems, MDS Sciex), fitted with a nano-electrospray ionization source (Proxeon, Odense, Denmark) using a 10 μm fused silica emitter tip (New Objectives, Woburn, MA, USA) and interfaced with an integrated LC system consisting of a Famos autosampler, SwitchOS II switching pump, and Ultimate micropump (LC Packings). Individual fractions containing peptides were injected onto a 300 μm × 5 cm C18 PepMap guard column (5 μm, 100A; LC Packings), resolved using a 75 μm × 150 mm analytical column (3 μm, 100A; LC Packings), and eluted using an automated binary gradient (200 nL/min) from 100% buffer A (2% acetonitrile (ACN), 0.05% formic acid in H_2_O) to 40% buffer B (0.05% formic acid in 98% ACN) in 40 min, then from 40% to 80% buffer B for 5 min. MS time of flight (TOF) scans were acquired from m/z 400 to 1200 for one second with up to two precursors selected for MS/MS from m/z 100 to 1500 using information-dependent acquisition at 2.5 seconds per scan; rolling collision energy was used to promote fragmentation.

### Custom predicted tryptic peptide database

**Figure 1:**
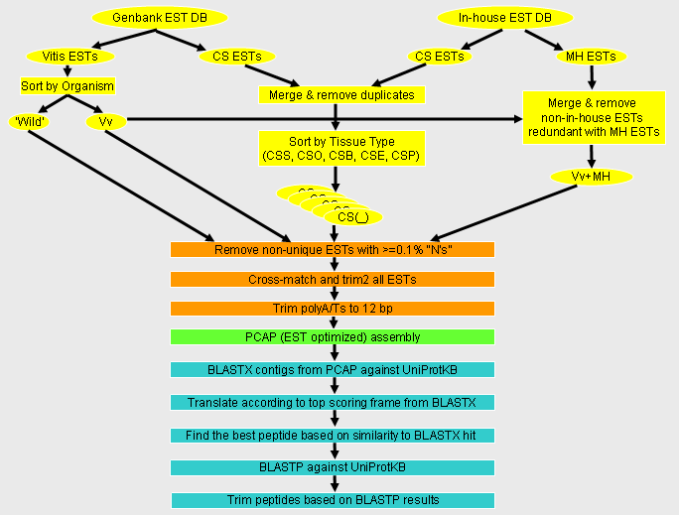
**Schema of the workflow in construction of the predicted peptide database based upon *Vitis *spp. ESTs**. Steps in EST selection (yellow), EST curation (orange), contig assembly (green), and translation, start methionine prediction, tryptic cleavage site prediction, and removal of predicted truncated N- and/or C-terminal peptides (blue) are shown. The in-house "GrapeGen" EST database was derived from the *V. vinifera *cv. Cabernet Sauvignon and Muscat Hamburg cDNAs [17]. Where a CS EST was duplicated between the Genbank and in-house databases, the Genbank EST was removed and the EST from the in-house database, containing the phred scores, was used for clustering. CS = Cabernet Sauvignon ESTs; MH = Muscat Hamburg ESTs; Vv = *Vitis vinifera*; Wild = *Vitis *spp. ESTs other than *V. vinifera*. A key to the codes used in 'Cluster ORF ID' and 'Protein Annotation' in Additional files 1 through 8 is presented in Additional file 10 and may be printed out as a quick reference when examining the iTRAQ results; tissue types (i.e. CS_) are described in Methods and Additional file 10.

A schema showing the pipeline for production of the predicted peptide database in support of this subsection is shown in Figure 1. All publicly available EST data for each *Vitis *species (AS, all sequences), including those from all *V. vinifera *(wine grape) cultivars, were downloaded in August 2007 as FASTA files from the National Center for Biotechnology Information (NCBI, Bethesda, MD, USA). These data were parsed on the basis of reported *Vitis *species of origin with the vast majority being from *V. vinifera *cultivars. Since we were specifically interested in studying the proteome in *V. vinifera *cv. Cabernet Sauvignon (CS) pericarp tissue, an additional, more rigorous approach to the parsing of the CS ESTs was carried out in order to reduce or eliminate the potential for subsequent assembly of paralogous CS sequences into invalid contigs, thereby striving to strengthen the validity of protein identification in our iTRAQ experiments. CS ESTs were obtained from the NCBI Genbank database or from an in-house EST project [17] and subdivided into the following categories based upon the reported source tissues for the cDNAs used for single pass sequencing: Whole berry including seed (CSB), berry without seed (pericarp, CSP), skin without seed or flesh (exocarp, CSE), seed only (CSS), and other tissues (CSO) including leaf, flower, tendril, and root. Because the in-house ESTs were also present in the NCBI Genbank database, the corresponding entries in Genbank were removed since the Genbank entries do not have sequence quality (phred [18]) scores. The following files containing EST data comprised each of the above mentioned groups: VV (VV.fasta representing all *V. vinifera *ESTs including in-house ESTs); WS (WS.fasta including ESTs from all available wild species, *V. aestivalis*; *V. cinerea *× *V. riparia*; *V. cinerea *× *V. rupestris*; *Vitis *hybrid (species not indicated in Genbank); *V. labrusca*; *V. pseudoreticulata*; *V. riparia*; *V. rotundifolia*; *V. shuttleworthii*), CSO (Bud.fasta; Flower_leaf _root.fasta; Leaf_blade.fasta; Petiole.fasta; Root.fasta; Flower-Pre-bloom.fasta; Inflorescence_including_flowers.fasta; Stem.fasta; Nectary_of_flowers.fasta; Flower_Bloom.fasta; Leaf.fasta; Inflorescence.fasta), CSS (Seed.fasta), CSP (Pericarp.fasta; Fruit_with_seeds_removed.fasta; Fruit_without_seeds.fasta), CSE (Fruit_skin.fasta), and CSB (Berry.fasta; Fruit.fasta).

Sequences were processed using cross_match (minmatch 12, penalty -2, minscore 20; ) and trim2 (G. Williams, ) in order to remove vector sequences as well as ambiguous nucleotides at the sequence ends. To successfully perform the above cleanup analyses, phred quality scores were used where available; otherwise, 'place-holder' quality scores were generated for any sequences for which no phred scores were available, as was the case for most of the ESTs in Genbank. Place-holder quality scores were also used later in the cluster assembly process as discussed in more detail, below. Following the cross_match and trim2 processing, the sequences were further trimmed using Perl scripts designed in-house to eliminate known invalid sequences (e.g. microbial sequences, simple sequence repeats) and trim polyA/T tails, if present in a given sequence. PolyA/T stretches were limited to 12 bp in order to prevent subsequent chimeric contig assembly based on those repeats. If polyA was followed by a > 30 bp stretch of AC, AT, GC, or GT repeats, the polyA stretch was trimmed to 12 bp and all sequence 3' to this was discarded; if polyT was preceded by a > 30 bp stretch of AC, AT, GC, or GT repeating sequence, the polyT stretch was trimmed to 12 bp and all sequence 5' to this was discarded. If polyA started at least two thirds of the EST sequence length, it was trimmed to 12 bp; if polyT started at less than one third of the EST sequence, it was trimmed to 12 bp. Any part of a sequence that started or ended with > 30 bp of repeats of AC, AT, GC, or GT was deleted. If a sequence started or ended with 'N's (indicating ambiguous base calls), the 'N's were deleted and the corresponding quality scores were also removed.

To better ensure that contig assemblies were based on high quality nucleotide sequence data, percent 'N' (ambiguous base call) content was determined for each sequence. If the percentage was > 0.3 (i.e. four or more 'N's per 1000 bp), the flanking 100 bp regions where scanned for 'N's and, if present, were trimmed to exclude the 'N's, thereby lowering the total 'N' percentage. Sequences shorter than 200 bp were trimmed to the first and last occurrences of an 'N'. For resulting sequences longer than 50 bp, the 'N' percentage was recalculated and, if still > 0.3%, a record of the sequence was made. Each of these sequences was then compared with other sequences in a combined dataset using BLASTN to determine its uniqueness. If a given sequence was already represented in the dataset by another sequence with a lower 'N' content, the sequence in question was eliminated.

The curated sequence datasets were next clustered using PCAP software [19] with parameters of 95% overlap identity and 60 bp overlap length [17]. PCAP was used instead of CAP3 in order to take advantage of parallelized processing. Parallelization provided the ability to distribute each dataset assembly workload across 100 CPUs for significantly faster processing time. The PCAP assembly program was modified and recompiled with EST_flag set at 1 (the default is 0, which indicates genomic reads). The PCAP assembly step was followed by a series of post-assembly steps (bdocs -y 100 -z 0, bclean -y 100 -w 1, bcontig -y 100 -p 95, bconsen -y 100 -z 0 -p 95, bform -y 100). We performed two clustering permutations in order to test the effects of database design on peptide identification using our iTRAQ data. First, we clustered all sequences together to create the "AS" database, including WS, VV, and all CS_ files; all sequences were weighted evenly. Second, CSB, CSE, CSP, CSO, CSS, WS, and VV (including CS sequences) were clustered separately with higher weighting (place-holder scores) placed on CS sequences in the VV build and the original phred scores retained for the in-house CS sequences. Weighting was accomplished by assigning higher quality scores such that when polymorphisms were encountered by PCAP in an assembly, preference was given for selection of the CS nucleotide for the resulting contig. Following assemblies, the generated contigs and singletons were merged into one file for each dataset (AS, CSB, CSE, CSP, CSO, CSS, VV, WS). Any sequences longer than 2500 bp were suspected to be chimeric, so they were parsed to a separate file, translated in all 6 frames, and peptides with a minimal size of 80 amino acids before a predicted stop codon were submitted to a BLASTX search against the nr database. The resulting multiple peptides predicted within long contigs were coded with "LC", as well as with "F" for the translational frame, with the frame number (either positive for forward or negative for reverse) and the peptide number designated from among the multiple peptides (separated by a period from the frame number).

A BLASTX analysis was next performed on each contig and singleton sequence against the nr database in order to identify the best frame for subsequent *in silico *translation. The frame identified via BLASTX analysis was then used to generate the predicted ORF (i.e. amino acid translation) for a given contig or singleton. In order to further curate predicted ORFs, each was subjected to *in silico *cleavage at any 'unknown' amino acid ('X') or stop codon and then compared to a similarly generated list of peptides from the corresponding best scoring protein sequence identified in the BLASTX search. The 'best peptide' was then identified in the translation frame as the peptide with an exact match to the BLASTX peptide. If no such peptide could be identified, the longest peptide generated by *in silico *cleavage of the sequence at each occurrence of an unknown amino acid and/or stop codon was used. All sequences which resulted in "no hit found" in the BLASTX results were subsequently translated in all six frames and appended to the end of the 'best peptide' file. In all cases where a six-frame translation was applied, the resulting peptides (designated as 'NH' in the database) were cleaved *in silico *at every unknown amino acid and/or stop codon and only those sequences 80 amino acids or longer were kept.

The resulting list of 'best peptides' for each of the sequences was then subjected to BLASTP analysis using the UniProtKB database in order to determine the sequence identity. The five highest BLASTP hits for each query sequence were aligned using an in-house Perl script to identify putative N- and/or C-termini. If no consensus site could determined for an N- or C-terminus via alignment with similar sequences, then these sequences were trimmed at the tryptic digestion site nearest to the ends of the predicted ORF to eliminate potentially truncated predicted tryptic peptides from the database. The parameters programmed into the scripts included: 1) BLASTP e-values, where E = H indicates a less significant hit (> 1e-05) and E = L indicates a stronger hit (E ≤ 1e-05), 2) the difference in length of the *Vitis *query sequence versus each of the top five subject sequences, and 3) the length of the exact match of amino acids to the top hit. These parameters improved automation of accurate predictions of methionine (M) sites and identification of likely full-length amino acid sequences without requiring manual inspection of the BLASTP results. Sequences identified with a predicted methionine at the N-terminus were coded with '(M)'. Determinations of C termini were done based on a small range cutoff (± 2) of amino acids between the stop codon in each top hit and the predicted stop codon in each corresponding *Vitis *ORF; if the difference was greater than two amino acids and deemed unclear, the C-terminal end of the predicted ORF was trimmed at the nearest upstream tryptic cleavage site.

The detailed process, above, was applied to each data set individually (AS, WS, VV, CSO, CSS, CSP, CSE and CSB). In preparation for the merger of the datasets, the CS tissue-specific sequences were analyzed for uniqueness based on comparing each sequence to every other sequence and discarding all shorter sequences for each exact match. Once all CS duplicate sequences were removed, this dataset was merged with the remaining two sets, VV and WS. This final set consisting of all of the sequences was then subjected to yet another uniqueness test where each sequence was compared to every other sequence but this time CS sequences where intentionally not removed, even if an exact duplicate existed in either the VV or the WS set and was of greater length. This allowed for preferential retention of the CS sequences in order to keep information about the tissue of origin of a detected protein. Out of a total of 113243 sequences submitted to the uniqueness test, 52394 were identified with CS duplicates present due to the preferential retention of those sequences. From the resulting sequences, only those that started with a predicted methionine were then submitted for SignalP analysis  and processing which allowed for the identification and ultimate trimming of signal peptides giving rise to predicted mature protein sequences. Those trimmed sequences that had a predicted cleavable targeting signal were coded '(SP)'. After removal of the predicted signal peptides, a final uniqueness test was performed.

### Analysis of MS/MS data

iTRAQ MS/MS data were analyzed using ProteinPilot software v. 2.0.1 (Applied Biosystems) for both tryptic peptide identification and quantification. The peptides and corresponding relative abundances were obtained in ProteinPilot using a confidence cutoff (called a 'Prot Score') of > 1.3 (> 95%). Database searching for each sample was done on predicted tryptic peptide sequence data using either the MSDB database (Release 20063108, Hammersmith Campus of Imperial College London) or in-house databases (Vitis_spp_ORF_db_v1.0, untrimmed, or AS databases). Annotations and annotated protein names indicated in ProteinPilot output files were coded to indicate several parameters specific to the ORF identified as well as the EST or contig from which the ORF sequence was predicted.

iTRAQ data representing the four ripening initiation stages in each of the three exocarp samples (2004, 2005-1, 2005-2) were combined into a single tab delimited file. Likewise, iTRAQ data representing each of the three mesocarp samples (2004, 2005-1, 2005-2) were combined into a second tab delimited file. Duplicate entries among exocarp or mesocarp files were identified using an in-house script in the R environment with 'Custom ORF ID' as the search string. Then, ratiometric data at each of the three comparisons using 'green' as the reference stage (i.e. pink/green; red/green; purple/green) were averaged prior to export for cluster analyses. Entries with the same name but different template cDNA sources were not averaged since these may represent isoforms from different source tissues and/or cultivars. We chose to cluster all proteins detected in the exocarp or mesocarp in order to capture all information on expression patterns detected, without restricting our analyses to only those proteins that were replicated amongst the individual exocarp or mesocarp files. K-means clustering into four partitions was carried out on ratiometric data for the exocarp and mesocarp files separately using MultiExperiment Viewer (MeV) software (The Institute for Genome Research; ). We used a 1.5-fold threshold for biological significance which was validated by consistencies between trends in protein expression presented here as increasing or decreasing with corresponding patterns of gene expression identified in previous publications (see Figure 4 legend for citations).

## Results

### Protein detection and annotation

In order to strengthen the ability of MS/MS spectra annotation software such as ProteinPilot to accurately identify peptide sequences in complex total protein samples, we performed weighted clustering on a large EST collection generated from *Vitis *spp. to create the most cultivar-specific peptide map database possible for Cabernet Sauvignon. In addition, we created Perl scripts in order to find the N and C termini in translated ORFs or otherwise to carry out trimming at known tryptic digestion sites. This was done to help decrease the number of incorrectly annotated, non-tryptic (i.e. truncated) N- and/or C-terminal peptides incorporated into protein abundance quantitations. To further increase the number of identifiable peptides, our in-house scripts searched all of the predicted ORFs designated as containing a likely start methionine using SignalP to identify and remove putative N-terminal signal peptides, resulting in a database containing mature protein sequence data for the predicted full length proteins.

To assess whether the custom tryptic peptide database improved protein discovery, we examined the number of high confidence proteins and peptides identified by ProteinPilot using this database versus a common approach to annotation, a search of the MSDB. We performed this analysis on two iTRAQ data sets, mesocarp 2005-1 and exocarp 2005-1, derived from four stages of Cabernet Sauvignon berries at ripening initiation. At a confidence level of 95% (p < 0.05), 1424 proteins were identified in the mesocarp using the custom database, whereas only 1184 proteins were identified in a search of the same data set using the MSDB (Table 1). At a confidence level of 95%, 1493 proteins were identified in the exocarp using the custom database, whereas 1390 proteins were identified in a search of the same data set using the MSDB (Table 1). These results indicate that in these two iTRAQ data sets, the use of the custom tryptic peptide database improved high confidence protein discovery by 20.2% and 7.4%. The number of high confidence peptides detected using the custom database was 1.9-fold and 1.8-fold higher in mesocarp and exocarp, respectively, in comparison to searches of the same iTRAQ data sets using the MSDB (Table 1). The greater difference in the number of high confidence peptides versus proteins detected using the custom database in comparison to the MSDB indicates that the most important impact of implementing the custom database was that it afforded the identification of more high confidence peptides per protein than could be achieved using the MSDB.

**Table 1:** Comparison of numbers of high confidence peptides and proteins detected by ProteinPilot based on the amino acid sequence database searched.

**Sample**	**Database searched**	**Proteins detected**	**Distinct peptides detected**
2005 mesocarp	MSDB^a^	1184	5679
2005 mesocarp	Custom *Vitis *DB with weighting^b ^and trimming^c^	1424	10793
			
2005 exocarp	MSDB	1390	6915
2005 exocarp	Custom *Vitis *DB with weighting and trimming	1493	12945
2005 exocarp	Custom *Vitis *DB with trimming but no weighting	1447	12956
2005 exocarp	Custom *Vitis *DB with weighting but no trimming	1382	8845

^a^Release 20063108, Hammersmith Campus of Imperial College London

^b^EST clustering weighted higher scores for ESTs derived from Cabernet Sauvignon in order to avoid the potential loss of SNPs specific to this cultivar during consensus sequence determination by the PCAP software

^c^Predicted tryptic peptides that were determined to likely be truncated were trimmed and removed from the custom peptide database

The effects of weighting and trimming on high confidence peptide and protein detection were analyzed using the 2005 exocarp iTRAQ data set. We performed a second build of all *Vitis *spp. ESTs using PCAP. For the purposes of this comparative analysis, all ESTs in this second build were equally weighted for consensus sequence determination using an arbitrary phred score; no higher weighting of Cabernet Sauvignon-derived ESTs was used. This unweighted *Vitis *EST database was then used to generate a second predicted tryptic peptide database, including trimming of end-truncated peptides, as before. The number of high confidence peptides and proteins detected were similar using the ORF databases created from the weighted versus the unweighted EST databases (Table 1), suggesting that the effect of simple nucleotide polymorphisms (SNPs) among the *Vitis *spp. ESTs is ultimately negligible at the level of peptide identification via MS/MS in grapevine. The effectiveness of trimming predicted end-truncated peptides, on the other hand, was evident in the increased number of peptides identified (Table 1) by ProteinPilot in searches of the trimmed (default) ORF database versus a third, untrimmed custom *Vitis *ORF database that we created for this comparison by omitting the trimming step shown in Figure 1; both of these databases were weighted for Cabernet Sauvignon ESTs in the original PCAP builds. These findings indicate that automated searching for the best predicted methionine start site and removing truncated N- and/or C-terminal tryptic peptide sequences in predicted ORFs based upon EST data increases the ability of MS/MS spectra annotation software to identify peptide sequences with high statistical confidence.

### Technical and biological replication of expression ratios

Proteins identified in iTRAQ data sets from two technical replicates were analyzed. Two 100 μg aliquots from the same total protein sample, exocarp 2004, were separately labeled with iTRAQ reagents, subjected to nanoLC-MS/MS, and the custom predicted tryptic peptide database was searched with the resulting spectra using ProteinPilot. Only proteins that were detected in each of the four ripening initiation stages were retained for the comparative analysis. In-house custom scripts written in R language [20] were employed to detect protein entries that were duplicated in the two ProteinPilot output files representing the technical replicates, using 'Custom ORF ID' as the search string. Out of a total of 1741 proteins detected in both iTRAQ data sets, 507 or 29% of these proteins were detected in both files (Additional file 1), whereas the remainder were unique to either technical replicate with 547 proteins in one file and 687 proteins in the other. We were interested in estimating consistency in trends in expression data along ripening initiation for each replicated protein entry that we detected (Additional file 1). We determined that strict estimation of replicability via Pearson's correlation coefficient or concordance correlation coefficient analyses of expression ratios did not provide relevant quantitative information (data not shown), since even small deviations in expression ratios can result in poor correlative data. We considered that identifying consistent trends in protein accumulation above an arbitrary cutoff such as 1.5-fold was most relevant for the purposes of discovering protein candidates for hypothesis formulation related to ripening control. As an alternate approach to correlation estimation, a replication scoring system was devised, whereby expression ratios identified by manual inspection as differing by less than log_2_(0.3) for each duplicate protein entry for at least two of the three ratios received a score of 1, expression ratios differing by between log_2_(0.3) and log_2_(0.6) for at least two of the three ratios received a score of 2, and expression ratios differing by log_2_(0.6) or greater for at least two of the three ratios received a score of 3. The mean replication score for the 507 duplicated protein entries in the technical replicates was determined to be 1.7 ± 0.7 (Additional file 1); 17% of the protein entries received a score of 3. These data indicate that if a 1.5-fold change or greater is considered as biologically relevant, over 80% of duplicate protein entries but only approximately one quarter of all proteins detected in two technical replicates would be expected to be identified as significantly changing along ripening initiation with similar quantitative trends, based on the technical replicates analyzed here.

Proteins identified in iTRAQ analyses of two biological replicates, exocarp 2004 and exocarp 2005-1 were next analyzed in an identical manner to the two technical replicates. Out of a total of 2187 proteins detected in the two iTRAQ data sets, 718 or 33% of these proteins were detected in both files (Additional file 2), whereas the remainder were unique to either biological replicate with 733 proteins in one file and 736 proteins in the other. The same replication scoring system was implemented for proteins detected in both iTRAQ data sets (Additional file 2) as was done for the technically replicated proteins (Additional file 1), above. The mean replication score for the 718 proteins common to both biological replicates was determined to be 1.6 ± 0.7 (Additional file 2); 12% of protein entries received a score of 3.

To further explore the biological significance of replicability analyses, proteins common to either the technical replicates (Additional file 1; 507 proteins) or the biological replicates (Additional file 2; 547 proteins) were compared and it was determined that 343 protein entries were common to the technical replicates and the biological replicates, i.e. were detected in all three data sets analyzed for replication (Additional file 3); we considered this approach as valid since all data analyzed here were derived from the exocarp. The technical and biological replication results indicate that the 'shotgun' LC-MS/MS approach employed here with iTRAQ-labeled exocarp total proteins is capable of repeatedly identifying approximately one third of the total number of proteins, whether they are technical or biological replicates.

### Expression profiling of proteins along grape berry ripening initiation

**Figure 2:**
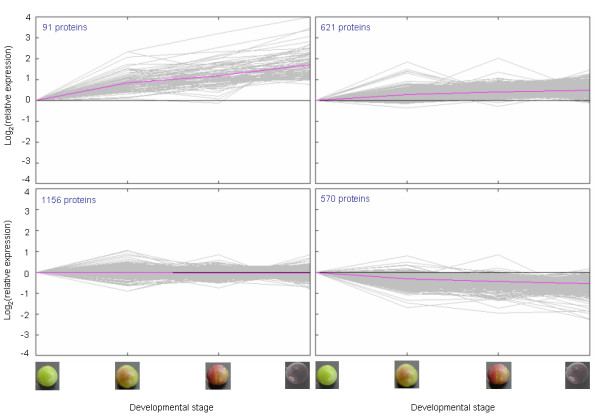
**K-means cluster analysis of expression data for exocarp proteins**. Four partitions were used to classify proteins that were increasing strongly (top left panel), increasing gradually (top right panel), not changing significantly (bottom left panel), or decreasing (bottom right panel) along ripening initiation. Ripening initiation stages corresponding to the expression data are depicted by the photographs on the two lower x-axes. X-axes on the lower two panels also correspond identically to the top two panels. Y-axes in the left two panels correspond identically to the right two panels. Numbers of proteins are shown in the top left corner of each panel.

Four clusters were generated for the exocarp in which trends in protein accumulation were increasing strongly (91 proteins), increasing gradually (621 proteins), not changing significantly (1156 proteins), or decreasing (570 proteins), respectively, from the green through the fully turned ripening initiation stages (Figure 2). Additional file 4 lists proteins by cluster number along with corresponding log_2_-transformed ratiometric data for each protein entry. Ratiometric data were calculated relative to the green stage for each of the pink/turning, red, and purple/fully turned stages. Several protein isoforms significantly increasing along ripening initiation were identified with annotated functions in anthocyanin flavonoid biosynthesis and storage (e.g. flavanone-3-hydroxylase, flavonoid-3'-hydroxlase, flavonoid-3'5'-hydroxylase and its associated cytochrome b5 protein, anthocyanin synthase, anthocyanidin 3-O-glucosyl transferase, putative anthocyanin *O*-methyltransferase), defense (e.g. pathogenesis-related protein 4, thaumatin (VvTL1)), and cell expansion (e.g. expansin, aquaporin). Conversely, several components of the photosynthetic machinery were identified as decreasing greater than 1.5-fold along ripening initiation, which is consistent with a reduction in photosynthesis at this period of berry development.

We mined the exocarp data (Additional file 4) for proteins that were increasing in abundance relative to the green stage and annotated as enzyme or transporter components of pathways leading to hypothesized regulators of ripening initiation in grapes, ABA, glucose, and BR. A putative LytB (IspH) protein (Q9FEP0; *Adonis aestivalis *cv. palaestina) increased 1.6-fold in abundance and is responsible for the last step of the plastidic pathway to isopentenyl diphosphate (IDP), leading in part to the production of the plant hormones, ABA and gibberellic acid. Other proteins of this MEP pathway were also detected but only as slightly increasing in abundance along ripening initiation. An isopentenyl-diphosphate δ-isomerase I protein was detected as increasing 2-fold along ripening initiation (Q39472; *Clarkia breweri*; Q6EJD1; *Pueraria montana*); this enzyme controls a major early step in isoprenoid biosynthesis and is likely localized to the chloroplast. We identified one component specific to the ABA biosynthetic pathway, a protein similar to violaxanthin de-epoxidase from tea (Q8S4C2; *Camellia sinensis*), which was stably expressed along ripening initiation. Cytosolic IDP, potentially also formed in the plastid and exported to the cytoplasm, is incorporated in the biosynthesis of BRs. A putative grapevine ortholog to a BR biosynthetic protein from pea, PsLKB, (Q9ATR0; *Pisum sativum*), was identified as increasing 1.6-fold, peaking at the third (red) stage tested. A grapevine hexose transporter (VvHT6; Q4VKB3) was identified as increasing 1.5-fold, which is consistent with hexose accumulation during ripening initiation. Few proteins annotated with signal transduction functions were detected in any of the four clusters that could hypothetically be involved in ABA, glucose, and/or BR signaling. Abscisic stress response protein (VvASR; Q94G23) was previously implicated in cross-talk between ABA and glucose signaling [21]; expression data were variable, with some isoforms increasing up to 4-fold along ripening initiation and others showing stable expression. A putative pirin protein of unknown function increased 4.5-fold along ripening initiation. A putative ortholog to the *Malus *spp. TTG1 WD40 repeat protein (Q9M610), which has previously been implicated in Arabidopsis in the regulation of anthocyanin biosynthesis [22], was detected as increasing, indicating that VvTTG1 could play a similar role in the grape exocarp; a caveat is that the confidence level for VvTTG1 unique peptide detection was less than 95%. Curiously, we did not identify peptides representing VvMYBA1, which is highly expressed at the transcriptional level during ripening initiation and encodes a transcription factor previously demonstrated to positively regulate anthocyanin biosynthesis genes in grape [23].

**Figure 3:**
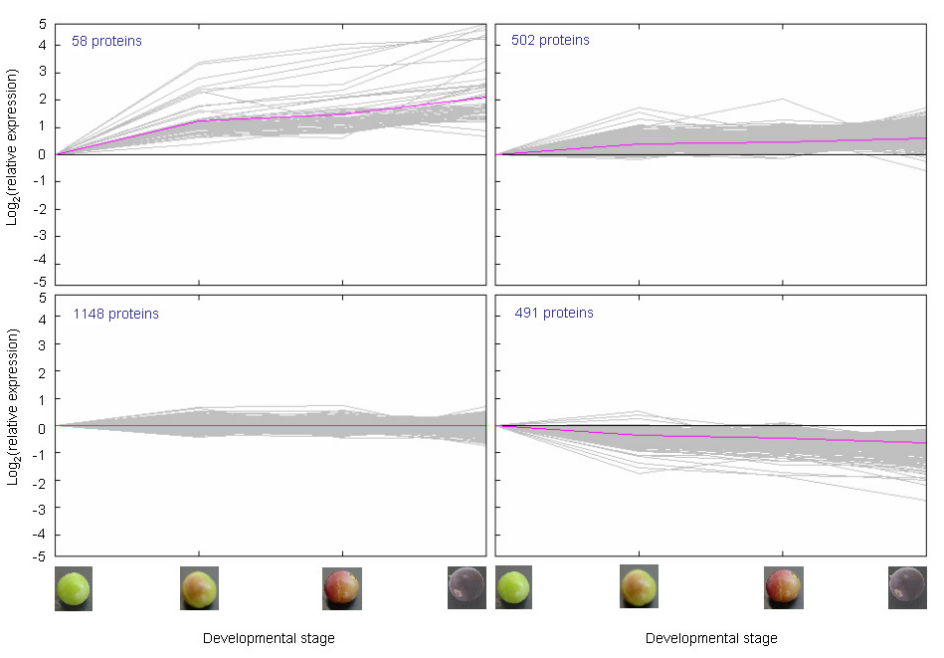
**K-means cluster analysis of expression data for mesocarp proteins**. Four partitions were used to classify proteins that were increasing strongly (top left panel), increasing gradually (top right panel), not changing significantly (bottom left panel), or decreasing (bottom right panel) along ripening initiation. Ripening initiation stages corresponding to the expression data are depicted by the photographs on the two lower x-axes. X-axes on the lower two panels also correspond identically to the top two panels. Y-axes in the left two panels correspond identically to the right two panels. Numbers of proteins in each cluster are shown in the top left corner of each panel.

Four clusters were generated for the mesocarp in which trends in protein accumulation were increasing strongly (58 proteins), increasing gradually (502 proteins), not changing significantly (1148 proteins), or decreasing (491 proteins), respectively, from the green through the fully turned ripening initiation stages (Figure 3). Additional file 5 lists proteins by cluster number along with corresponding log_2_-transformed ratiometric data for each protein entry, relative to the green stage. Similar to the exocarp data, we identified > 2-fold accumulation of several protein isoforms annotated with functions in cell enlargement (e.g. expansins, aquaporins), fruit softening (e.g. polygalacturonase, pectate lyase) and defense (e.g. pathogenesis-related proteins) concomitant with a significant reduction in proteins inhibitory to fruit ripening (e.g. polygalacturonase-inhibiting protein).

We mined the mesocarp data (Additional file 5) for proteins annotated as enzyme or transporter components of pathways leading to ABA, glucose, and brassinosteroid accumulation. We detected VvNCED2 (9-cis-epoxycarotenoid dioxygenase 2; Q5SGD0) increasing over 2-fold along ripening initiation, which represents expression of a key committed step specifically in ABA biosynthesis in the plastid [24]. A protein annotated as ABA glucosyltransferase increased 1.25-fold along ripening initiation in the mesocarp. As in the exocarp, an isopentenyl-diphosphate δ-isomerase I was detected as increasing 2-fold along ripening initiation (Q6EJD1; *Pueraria lobata*), plus, a putative cytosolic isoform was detected as stably expressed (*Nicotiana tabacum*; Q9AVG7) [25]. Farnesyl diphosphate synthase, a cytoplasmic enzyme leading to the BR biosynthetic pathway via squalene biosynthesis, was detected in the mesocarp as increasing 1.4-fold along ripening initiation. A putative grapevine ortholog to the BR biosynthetic protein in pea, PsLKB, (Q9ATR0; *Pisum sativum*), and in cotton, GhDWARF1 (Q2QCX8; *Gossypium hirsutum*), was detected as increasing 1.4-fold. The hexose transporter, VvHT6, was detected as increasing 2-fold, similar to its expression pattern that was detected in the exocarp. We mined the mesocarp data further to identify proteins annotated with signal transduction functions. No plasma membrane receptor candidates were identified as increasing but several were identified as not changing or decreasing along ripening initiation, most notably a receptor-like kinase similar to PERK1-like protein from rice (Q6ZIG4) that decreased 1.5-fold at the onset of pigment accumulation to 2.5-fold at the purple stage; this PERK1-like protein was also identified as significantly decreasing in the exocarp (Additional file 4). The same pirin protein identified in the exocarp was similarly detected as increasing in the mesocarp along ripening initiation. Inconsistent iTRAQ data were obtained for VvASR in the mesocarp with isoforms showing an increase, stable expression, or strong down-regulation.

**Figure 4:**
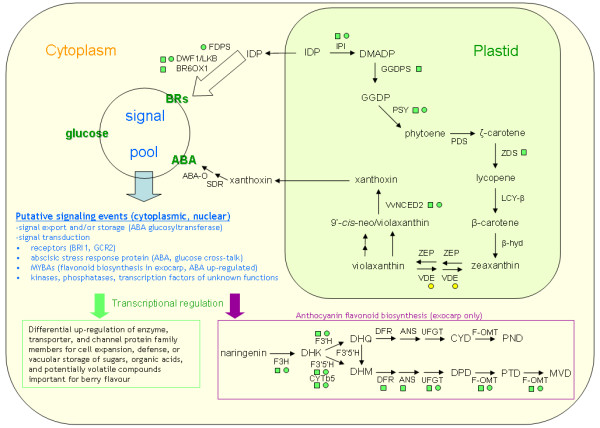
**Model showing transcript (squares) and protein (circles) expression trends annotated with functions in ABA, BR, and anthocyanin biosynthesis in the grape berry pericarp at ripening initiation**. Transcript accumulation data are based on previous studies [26,27,35-37], whereas all protein accumulation data presented here are new findings for grape berries. Green indicates an increase in transcript or protein abundance during ripening initiation. Yellow indicates no significant change in protein accumulation. ABA-O = abscisic aldehyde oxidase; ANS = anthocyanin synthase; BR6OX1 = brassinosteroid-6-oxidase; BRI1 = receptor-like kinase, brassinosteroid insensitive 1; β-hyd = β-carotene hydroxylase; CYD = cyanidin; CYTb5 = cytochrome b5; DFR = dihydroflavonol reductase; DHK = dihydrokaempferol; DHM = dihydromyricetin; DHQ = dihydroquercitin; DMADP = dimethylallyl diphosphate; DPD = delphinidin; DWF1 = dwarf 1; F-OMT = putative anthocyanin flavonoid *O*-methyltransferase; FDPS = farnesyl diphosphate synthase; F3H = flavonoid-3-hydroxylase; F3'H = flavonoid-3'-hydroxylase; F3'5'H = flavonoid-3'5'-hydroxylase; GCR2 = G protein-coupled receptor 2; GGDP = geranylgeranyl diphosphate; GGDPS = geranylgeranyl diphosphate synthase; IDP = isopentenyl diphosphate; IPI = isopentenyl diphosphate isomerase; LCY-β = lycopene-β-cyclase; MVD = malvidin; NCED = 9-cis-epoxycarotenoid dioxygenase; PDS = phytoene desaturase; PND = peonidin; PSY = phytoene synthase; PTD = petunidin; SDR = short-chain alcohol dehydrogenase/reductase; VDE = violaxanthin de-epoxidase; ZDS = ζ-carotene desaturase; ZEP = zeaxanthin epoxidase.

A custom script written in the R environment was used to search for proteins common to the combined exocarp and mesocarp files (Additional files 4 and 5) using 'Custom ORF ID' as the search string. Protein isoforms detected in common between exocarp and mesocarp (1147 in total) are shown in Additional file 6. Protein isoforms detected only in exocarp (1149 in total) are shown in Additional file 7. Protein isoforms detected only in mesocarp (905 in total) are shown in Additional file 8. Isoforms of anthocyanin flavonoid biosynthetic proteins were only detected in the exocarp, which is consistent with induction timing and tissue localization of these pigments during ripening initiation and demonstrates the efficacy of our separation of exocarp and mesocarp for proteomic analyses. Additional file 9 shows the source tissues and number of unique high confidence peptides per protein for those proteins indicated here in the Results section and in Figure 4.

## Discussion

The iTRAQ data obtained with exocarp and mesocarp total proteins confirmed previous ratiometric transcript abundance data for key components of ABA and BR biosynthesis, as well as the influx and accumulation of sugars during ripening initiation (Figure 4). We confirmed for the first time the strong accumulation of several components of anthocyanin biosynthesis at the protein level, including a putative anthocyanin *O*-methyltransferase. The detection of VvNCED2 accumulation confirmed previous real-time RT-PCR data [26] and further supports a role specifically for this NCED family member in ABA biosynthesis in the mesocarp during berry ripening initiation. Based on the sensitivity limitations to the shotgun proteomic technique employed here, we cannot conclude that VvNCED2 accumulation, activity, and, consequently, ABA biosynthesis are localized to the mesocarp; VvNCED2 may be expressed in the exocarp but we did not detect it. The previous detection of ζ-carotene desaturase transcripts in the exocarp [27] argues against a model in which ABA is synthesized in the mesocarp and transported to the exocarp whereupon it activates, in part, anthocyanin biosynthesis. If the primary site of ABA production in the developing fruit is the seed, however, and ABA transported from the seed to the surrounding pericarp is an early signaling event initiating ripening, as we have previously hypothesized [26], it is reasonable that the first tissue in the pericarp in which ABA is synthesized is the mesocarp, given its closer proximity to the seed than the exocarp. It remains to be determined whether a gradient in ABA biosynthesis during early ripening stages proceeds from the seed through the mesocarp and, finally, in the exocarp. The presence and possible moderate accumulation of a putative ABA glucosyltransferase indicates that an attenuating mechanism for the ABA signal may operate during ripening initiation to control ABA homeostasis [28]. The accumulation of the pirin protein is also intriguing, given this protein's previously demonstrated interaction with G-protein alpha in Arabidopsis seeds and a potential role in modifying ABA action via negative feedback control [29].

We used a relatively new quantitative MS/MS-based approach, iTRAQ, to advance our understanding of differential protein expression underlying non-climacteric ripening initiation in grape berries. The iTRAQ approach offered several advantages over 2DGE methods for protein discovery, including increased detection sensitivity based on our findings reported here in comparison to previous reports on grape berry proteomics. By using strong cation exchange and reverse phase column microcapillary chromatography coupled with nanospray MS/MS detection with total protein extracts from grape berries, we were able to resolve three-fold or more proteins per sample than would be expected using 2DGE [11,12,14]. One current limitation to an MS-based proteomic approach with grapevine is that there are no finished genome sequence data for grapevine, although two projects [30,31] are undertaking assembly and annotation from which a high quality ORFeome database can eventually be derived. Although there are over 300,000 *Vitis *spp. ESTs deposited in Genbank, *V. vinifera *is a highly heterozygous species [31]; therefore, we considered that it might be important to weight builds of these ESTs via manipulation of phred scores so as to favor sequence data corresponding to our cultivar of interest, Cabernet Sauvignon, when SNPs were encountered by PCAP, where applicable for a given contig assembly. Although we determined that weighting ESTs to the genotype of interest for EST assembly provided no clear advantages in this study, we conclude that producing a tryptic peptide database targeted to *Vitis *sequences and including removal of predicted truncated peptides improved protein detection and annotation. Furthermore, our findings indicate that a tryptic peptide database based on finished Pinot Noir whole genome sequence data will be valid to implement for proteome studies with any *V*.*vinifera *cultivar or *Vitis *species, with the exception of cases where deletions have occurred in the Pinot Noir homozygous line [30].

We chose to not include genome sequence data available for *V. vinifera *cv. Pinot Noir [30,31] due to significant gaps in current assemblies and the potential for inaccurate automated gene predictions. Until grapevine genome sequence assembly and annotation are finished, we propose that the predicted ORF database presented here will be of value to the grapevine community in two significant ways. While gaps exist in the genome sequence assemblies, the protein database presented here may provide information for 'missing' proteins either not yet predicted from the Pinot Noir genome sequence data and/or other *Vitis *spp. not represented in the Pinot Noir genome sequence data, e.g. due to chromosomal deletions. Further, protein predictions based upon expressed sequences (ESTs) represent 'real' proteins, so our database could potentially be used to validate ORF predictions based on whole genome sequence data alone.

While iTRAQ labeling coupled with nanoLC-MS/MS proved overall to be an advance over 2DGE in sensitivity and quantitation, consistent detection of predicted protein sequences between technical or biological replicates from grape exocarp was limited. Consistency in trends in ratiometric data along ripening initiation for those proteins that were detected in replicate exocarp samples was further limited. Inconsistent ratiometric data for some proteins detected in both biological replicates may represent differences in expression due, for example, to variability in seasonal growing conditions and not technical variability. Nonetheless, limited replicable detection of proteins among biological samples for technical reasons is commonly encountered with liquid chromatography and MS-based proteomics [32] and could arise from variation in preparation of total proteins and/or iTRAQ-labeled peptides from sample to sample. Underlying the apparent variation arising from sample preparations are constraints on detection imposed by the mass spectrometer, both with respect to the dynamic range of the instrument and the nature of selections of peptides in the first MS by the MS software for export to the collision cell prior to amino acid detection via the second MS. Furthermore, our finding that two thirds of the replicated exocarp proteins were detected both in technical and biological replicates may have reflected the higher probability of detecting these mainly abundant, housekeeping-type proteins in any given total protein sample using a shotgun approach.

Over-sampling of abundant peptides in digested total protein samples is a limitation of the shotgun approach to quantitative proteomics [33] and likely precluded our ability to discover more proteins annotated with signal transduction functions that could regulate ABA, BR, and hexose responses during ripening initiation. In order to increase detection sensitivity of low abundance regulatory proteins using shotgun proteomics techniques, it will likely be helpful to isolate membrane and nuclear proteins separately from cytoplasmic proteins prior to digestions. Affinity chromatography of berry protein extracts using antibodies directed against abundant proteins such as thaumatin detected in both exocarp and mesocarp may also improve detection sensitivity for low abundance proteins by selectively removing these proteins prior to iTRAQ labeling steps. Similarly, advances in detection sensitivity in devices such as the Fourier transform ion cyclotron resonance MS (FTICR-MS) [34] should allow us to delve deeper into the grape berry proteome in order to better understand the molecular control of non-climacteric ripening in this species. Nonetheless, the searchable quantitative data presented here on over 3000 proteins detected in two separate berry tissues along four ripening initiation stages can serve as a framework public resource contributing towards our understanding of the dynamic grapevine proteome.

## Conclusion

We determined that a predicted peptide database can be derived from grapevine EST data using advanced clustering and trimming approaches and successfully implemented for quantitative proteome profiling. We demonstrated in grapevine that by implementing a predicted peptide database targeted to an organism of interest, a significant increase in the number of high confidence peptides identified and annotated from MS/MS data was gained in comparison to more commonly used MSDB searches. Furthermore, by using a shotgun quantitative proteomics approach in combination with a targeted predicted peptide database, we showed that greater numbers of high confidence peptides were detected than would be expected using 2D gel electrophoresis techniques. We verified for the first time at the protein level, quantitative expression patterns for components of isoprenoid and flavonoid metabolism important for grape compositional chemistry, as well as identified new signal transduction candidate proteins associated with non-climacteric ripening initiation in grape berries.

## Abbreviations

ABA: abscisic acid; BLAST: basic local alignment and search tool; BR: brassinosteroids; EST: expressed sequence tag; iTRAQ: isobaric tagging for relative and absolute quantitation; ORF: open reading frame; PCAP: parallel contig assembly program

## Authors' contributions

JL designed the experiments, carried out protein extractions and data analyses, assisted in designing the scripts for the predicted peptide database, and drafted the manuscript. ML designed the scripts and carried out programming to produce the predicted peptide databases presented here. DS carried out mass spectrometry and database searches using the iTRAQ MS/MS data. STL performed primary data analyses, assisted in drafting the manuscript, and was responsible for overseeing the project.

## Supplementary Material

Additional file 1 **Duplicated proteins and associated replication scores for exocarp 2004 technically replicated iTRAQ experiments.** Ratiometric data are given in log_2 _scale. Click here for file

Additional file 2 **Duplicated proteins and associated replication scores for exocarp 2004 and exocarp 2005-1 biologically replicated iTRAQ experiments.** Ratiometric data are given in log_2 _scale. Click here for file

Additional file 3 **Proteins identified in all of the three iTRAQ data sets analyzed (exocarp 2004 (two technical replicates) and exocarp 2005-1) and tested for replication in expression trends along ripening initiation.** Click here for file

Additional file 4 **Exocarp proteins listed for each K-means cluster along with corresponding log_2_-transformed ratiometric data for each protein entry.** Exocarp proteins from the 2004, 2005-1, and 2005-2 datasets were combined into a single file. Duplicate entries were identified using an in-house script in the R environment with 'Custom ORF ID' as the search string. Then, ratiometric data at each of the three comparisons using 'green' as the reference stage were averaged prior to export for K-means cluster analyses. Click here for file

Additional file 5 **Mesocarp proteins listed for each K-means cluster along with corresponding log_2_-transformed ratiometric data for each protein entry.** Mesocarp proteins from the 2004, 2005-1, and 2005-2 datasets were combined into a single file. Duplicate entries were identified using an in-house script in the R environment with 'Custom ORF ID' as the search string. Then, ratiometric data at each of the three comparisons using 'green' as the reference stage were averaged prior to export for K-means cluster analyses. Click here for file

Additional file 6 **Protein isoforms detected in common between the exocarp and mesocarp (Additional files 4 and 5, respectively).** A custom script written in the R environment was used to search for proteins common to the combined exocarp and mesocarp files using 'Custom ORF ID' as the search string. Click here for file

Additional file 7 **Protein isoforms detected only in exocarp samples.** Proteins excluded from Additional file 6 that were detected in the combined exocarp file (Additional file 4) are shown. Click here for file

Additional file 8 **Protein isoforms detected only in mesocarp samples.** Proteins excluded from Additional file 6 that were detected in the combined mesocarp file (Additional file 5) are shown. Click here for file

Additional file 9 **Protein source tissues and number of unique high confidence peptides per protein.** Proteins presented in the Results and Discussion sections as well as in Figure 4 are shown. The source tissue for each protein hit shown in Column A corresponds to a ProteinPilot output file, which can be downloaded at  The number of unique > 95% confidence peptides determined by the ProteinPilot software for each protein is shown in Column E. Ratiometric data shown in Columns G, H, and I are relative to the green stage (Column F) and are not log_2_-transformed. Click here for file

Additional file 10 **Reference key for coding terms used in Column A (Cluster ORF ID) and Column B (Protein Annotation) in Additional files 1 through 8.** The key is provided as a printable rapid reference for data mining in Additional files 1 through 8, as well as in the protein database available online, as shown in the Acknowledgments. Click here for file

## References

[B1] Perkins DN, Pappin DJC, Creasy DM, Cottrell JS (1999). Probability-based protein identification by searching sequence databases using mass spectrometry data. Electrophoresis.

[B2] Shilov IV, Seymour SL, Patel AA, Loboda A, Tang WH, Keating SP, Hunter CL, Nuwaysir LM, Schaeffer DA (2007). The Paragon algorithm: A next generation search engine that uses sequence temperature values and feature probabilities to identify peptides from tandem mass spectra. Mol Cell Prot.

[B3] Choudhary JS, Blackstock WP, Creasy DM, Cottrell JS (2001). Matching peptide mass spectra to EST and genomic DNA databases. Trends Biotech.

[B4] Gygi SP, Rist B, Gerber SA, Turecek F, Gelb MH (1999). Quantitative analysis of complex protein mixtures using isotope-coded affinity tags. Nature Biotech.

[B5] Islam N, Tsujimoto H, Hirano H (2003). Wheat proteomics: Relationship between fine chromosome deletion and protein expression. Proteomics.

[B6] Pierce A, Unwin RD, Evans CA, Griffiths S, Carney L (2007). Eight-channel iTRAQ enables comparison of the activity of 6 leukaemogenic tyrosine kinases. Mol Cell Prot.

[B7] Dunkley TPJ, Svenja H, Shadforth IP, Runions J, Weimar T, Hanton SL, Griffin JL, Bessant C, Brandizzi F, Hawes C, Watson RB, Dupree P, Lilley KS (2006). Mapping the Arabidopsis organelle proteome. Proc Nat Acad Sci USA.

[B8] Jones AME, Bennett MH, Mansfield JW, Grant M (2006). Analysis of the defense phosphoproteome of *Arabidopsis thaliana *using differential mass tagging. Proteomics.

[B9] Patterson J, Ford K, Cassin A, Natera S, Bacic A (2007). Increased abundance of proteins involved in phytosiderophore production in boron-tolerant barley. Plant Physiol.

[B10] Lund ST, Bohlmann J (2006). The molecular basis for wine grape quality – A volatile subject. Science.

[B11] Deytieux C, Geny L, Lapaillerie D, Claverol S, Bonneu M, Donèche B (2007). Proteome analysis of grape skins during ripening. J Exp Bot.

[B12] Giribaldi M, Perugini I, Sauvage F-X, Schubert A (2007). Analysis of protein changes during grape berry ripening by 2-DE and MALDI-TOF. Proteomics.

[B13] Castro AJ, Carapito C, Zorn N, Magné C, Leize E, Van Dorsselaer A, Clément C (2005). Proteomic analysis of grapevine (*Vitis vinifera *L.) tissues subjected to herbicide stress. J Exp Bot.

[B14] Sarry JE, Sommerer N, Sauvage FX, Bergoin A, Rossignol M, Albagnac G, Romieu C (2004). Grape berry biochemistry revisited upon proteomic analysis of the mesocarp. Proteomics.

[B15] Wang W, Scali M, Vignani R, Spadafora A, Sensi E, Mazzuca S, Cresti M (2003). Protein extraction for two-dimensional electrophoresis from olive leaf, a plant tissue containing high levels of interfering compounds. Electrophoresis.

[B16] Jain AK, Basha SM (2003). A capillary electrophoretic method for isolation and characterization of grape xylem proteins. Afr J Biotech.

[B17] Peng FY, Reid KE, Liao N, Schlosser J, Lijavetzky D, Holt R, Martínez Zapater JM, Jones S, Marra M, Bohlmann J, Lund ST (2007). Generation of ESTs in it *Vitis vinifera *wine grape (Cabernet Sauvignon) and table grape (Muscat Hamburg) and discovery of new candidate genes with potential roles in berry development. Gene.

[B18] Ewing B, Hillier L, Wendl MC, Green P (1998). Base-calling of automated sequencer traces using phred. I. Accuracy assessment. Genome Res.

[B19] Huang X, Wang J, Aluru S, Yang S-P, Hillier L (2003). PCAP: A whole-genome assembly program. Genome Res.

[B20] Development Core Team (2006). R: A Language and Environment for Statistical Computing. Vienna, Austria. http://www.R-project.org.

[B21] Çakir B, Agasse A, Gaillard C, Saumonneau A, Delrot S, Atanassova R (2003). A grape ASR protein involved in sugar and abscisic acid signaling. Plant Cell.

[B22] Walker AR, Davison PA, Bolognesi-Winfield AC, James CM, Srinivasan N, Blundell TL, Esch JJ, Marks MD, Gray JC (1999). The TRANSPARENT TESTA GLABRA1 locus, which regulates trichome differentiation and anthocyanin biosynthesis in Arabidopsis, encodes a WD40 repeat protein. Plant Cell.

[B23] Kobayashi S, Goto-Yamamoto N, Hirochika H (2004). Retrotransposon-induced mutations in grape skin color. Science.

[B24] Nambara E, Marion-Poll A (2005). Abscisic acid biosynthesis and catabolism. Annu Rev Plant Biol.

[B25] Phillips MA, D'Auria JC, Gershenzon J, Pichersky E (2008). The *Arabidopsis thaliana *Type I isopentenyl diphosphate isomerases are targeted to multiple subcellular compartments and have overlapping functions in isoprenoid biosynthesis. Plant Cell.

[B26] Lund ST, Peng FY, Nayar T, Reid KE, Schlosser J (2008). Gene expression analyses in individual grape (*Vitis vinifera *L.) berries during ripening initiation reveal that pigmentation intensity is a valid indicator of developmental staging within the cluster. Plant Mol Biol.

[B27] Grimplet J, Deluc LG, Tillett RL, Wheatley MD, Schlauch KA, Cramer GR, Cushman JC (2007). Tissue-specific mRNA expression profiling in grape berry tissues. BMC Genomics.

[B28] Xu Z-J, Nakajima M, Suzuki Y, Yamaguchi I (2002). Cloning and characterization of the abscisic acid-specific glucosyltransferase gene from adzuki bean seedlings. Plant Physiol.

[B29] Lapik YR, Kaufman LS (2003). The Arabidopsis cupin domain protein AtPirin1 interacts with the G Protein α-subunit GPA1 and regulates seed germination and early seedling development. Plant Cell.

[B30] The French-Italian Public Consortium for Grapevine Genome Characterization (2007). The grapevine genome sequence suggests ancestral hexaploidization in major angiosperm phyla. Nature.

[B31] Velasco R, Zharkikh A, Troggio M, Cartwright DA, Cestaro A, Pruss D, Pindo M, FitzGerald LM, Vezzulli S, Reid J, Malacarne G, Iliev D, Coppola G, Wardell B, Micheletti D, Macalma T, Facci M, Mitchell JT, Perazzolli M, Eldredge G, Gatto P, Oyzerski R, Moretto M, Gutin N, Stefanini M, Chen Y, Segala C, Davenport C, Demattè L, Mraz A, Battilana J, Stormo K, Costa F, Tao Q, Si-Ammour A, Harkins T, Lackey A, Perbost C, Taillon B, Stella A, Solovyev V, Fawcett JA, Sterck L, Vandepoele K, Grando SM, Toppo S, Moser C, Lanchbury J, Bogden R, Skolnick M, Sgaramella V, Bhatnagar SK, Paolo F, Gutin A, Peer Y Van de, Salamini F, Viola R (2007). A high quality draft consensus sequence of the genome of a heterozygous grapevine variety. PloS One.

[B32] Thelen JJ, Peck SC (2007). Quantitative proteomics in plants: Choices in abundance. Plant Cell.

[B33] Reinhardt TA, Lippolis JD (2008). Developmental changes in the milk fat globule membrane proteome during the transition from colostrum to milk. J Dairy Sci.

[B34] Zimmer JSD, Monroe ME, Qian WJ, Smith RD (2006). Advances in proteomics data analysis and display using an accurate mass and time tag approach. Mass Spec.

[B35] Symons GM, Davies C, Shavrukov Y, Dry IB, Reid JB, Thomas MR (2006). Grapes on steroids. Brassinosteroids are involved in grape berry ripening. Plant Physiol.

[B36] Deluc LG, Grimplet J, Wheatley MD, Tillett RL, Quilici DR, Deluc LG, Grimplet J, Wheatley MD, Tillett RL, Quilici DR (2007). Transcriptomic and metabolite analyses of Cabernet Sauvignon grape berry development. BMC Genomics.

[B37] Pilati S, Perazzolli M, Malossini A, Cestaro A, Demattè L, Fontana P, Dal Ri A, Viola R, Velasco R, Moser C (2007). Genome-wide transcriptional analysis of grapevine berry ripening reveals a set of genes similarly modulated during three seasons and the occurrence of an oxidative burst at veraison. BMC Genomics.

