# Supplementary material for: Generation of a predicted protein database from EST data and application to iTRAQ analyses in grape (Vitis vinifera cv. Cabernet Sauvignon) berries at ripening initiation
Source: BMC Genomics. 2009 Jan 26;10:50. doi: 10.1186/1471-2164-10-50 (PMC2637896; doi:10.1186/1471-2164-10-50)
Supplement: Additional file 10 — Reference key for coding terms used in Column A (Cluster ORF ID) and Column B (Protein Annotation) in Additional files 1 through 8. The key is provided as a printable rapid reference for data mining in Additional files 1 through 8, as well as in the protein database available online, as shown in the Acknowledgments. [file 1471-2164-10-50-S10.doc]

**Additional file 10. Reference key for coding terms used in Column A (Cluster ORF ID) and Column B (Protein Annotation) in Additional files 1 through 8.**

**Column A: Cluster ORF ID**

csb = Cabernet Sauvignon, whole berry (seed and/or pericarp not specified by depositing lab)

csp = Cabernet Sauvignon, pericarp (exoarp+mesocarp, not including seed)

cse = Cabernet Sauvignon, exocarp (berry skin) only

css = Cabernet Sauvignon, seed only

cso = Cabernet Sauvignon, non-berry (floral bud, floral inflorescence, floral nectary, pre-anthesis flower, post-anthesis flower, leaf, root, stem)

vv = *Vitis vinifera*, unique clones remaining from the PCAP assembly of all *Vitis vinifera* ESTs, excluding ws but including all cs ESTs

ws = wild species (*Vitis* spp.), derived from any tissue other than one from *Vitis vinifera*

gg = full-length sequence generated through the GrapeGen project [17]

gi = Genbank identifier

gb = Genbank nucleotide accession number

vvfl = *Vitis vinifera* full length protein deposited in Genbank, not based on whole genome sequence data; also note that in Excel, two ‘v’s adjacent to one another can sometimes appear to be a ‘w’, but this is vvfl

ctg = contig; references the PCAP build described in the Methods

F = translational frame, where a positive number indicates a forward frame and a negative number indicates a reverse frame; all frames were translated from each amino acid sequence and only the ones longer than or equal to 80 amino acids were retained. Note that the ‘F’code is only used with NH and LC contigs/singletons (see ‘Column B’, below).

**Column B: Protein Annotation**

The top two UniProtKB hits to each grapevine protein are shown, with the top hit shown to the left in each cell.

(M) = predicted methionine start codon (putative full-length ORF)

E=L = e-value≤10^-05^; higher confidence annotation

E=H = e-value>10^-05^; lower confidence annotation

NH = no hit

LC = long contig (see Methods for a detailed explanation)

SP = predicted N-terminal signal sequence removed

**Columns C through F (Additional files 1, 2, 4, 5): Ratiometric Data**

Data are log_2_-transformed ratios of pink/turning, red, or purple stages relative to the green stage of ripening initiation in Cabernet Sauvignon.
